# Designing a Positive Health Dialogue Tool for Adolescents and Young Adults: A Mixed Methods Study

**DOI:** 10.1111/hex.70158

**Published:** 2025-01-31

**Authors:** Cheyuan Liu, Liuyin Ji, Yujie Zhang, Jianrui Zhou, Qunyao Hu, Yaxin Wei, Chunyu Zhang, Fangzhou Liu

**Affiliations:** ^1^ School of Economics and Management Guangxi Normal University Guilin Guangxi China

Dear Editor,

I hope this message finds you well. I recently read the article “Designing a Positive Health Dialogue Tool for Adolescents and Young Adults: A Mixed Methods Study” published by your journal. First, I commend the authors, Marja van Vliet, Machteld Huber, and Sigrid van der Zanden, for their efforts and valuable insights in this research. The authors have designed a dialogue tool to assist young individuals aged 16–25 in navigating various health and vulnerability‐related issues, applicable across multiple domains, such as healthcare and social interactions [1]. However, I believe that the article may have certain limitations that warrant attention, and I would like to offer some suggestions for improving future research.

First, the sample size used in the study is relatively small (*N* = 118), particularly for the qualitative component (*N* = 36). This limited sample size may compromise the reliability and generalisability of the findings. Second, there is a notable gender imbalance within the sample, with 79% of participants being female, and the study focuses mainly on individuals in the Netherlands, which may limit the diversity of perspectives. Third, the study does not assess the long‐term effects of the dialogue tool, particularly concerning its sustained influence on health perception and resilience. Fourth, the data is primarily based on self‐reports from participants, which may introduce subjective biases [2]. Fifth, the discussion on the study's limitations is insufficient, lacking an in‐depth examination of how these limitations might impact the generalisability and applicability of the findings. For example, issues such as sample representativeness, the tool's applicability across diverse contexts, and the limitations inherent in qualitative data were not adequately addressed.

To enhance the quality of this study, several suggestions for improvement could be considered: First, the small sample size may affect the reliability and generalisability of the findings [3]. I recommend that future studies either increase the sample size or provide justification for sample size adequacy based on expected precision estimates [4, 5]. Second, to improve the external validity of the findings, I suggest conducting research with a more diverse sample that incorporates participants from different cultural contexts and includes a balanced gender ratio. Third, future studies could employ a longitudinal design to track participants over time, which would allow for the assessment of the sustained effects of the tool on health perception and resilience, thereby providing stronger empirical support and enhancing the long‐term applicability of the conclusions. Fourth, future research should incorporate objective data sources (e.g., medical records) to mitigate the influence of subjective biases. Fifth, I recommend a more comprehensive analysis in the conclusion regarding sample representativeness, the applicability of the tool in diverse contexts, and the limitations of qualitative data. This would help readers better understand the scope and limitations of the study's findings.

## Author Contributions

**Cheyuan Liu:** conceptualization, writing–review and editing. **Liuyin Ji:** methodology, data curation, formal analysis, writing–original draft. **Yujie Zhang:** investigation. **Jianrui Zhou:** validation. **Qunyao Hu:** formal analysis. **Yaxin Wei:** supervision. **Chunyu Zhang:** validation. **Fangzhou Liu:** supervision.

### Ethics Statement

The authors have nothing to report.

### Conflicts of Interest

The authors declare no conflicts of interest.

## Data Availability

The authors have nothing to report.
